# Mid-Term Surgical Outcomes of T-Hook, 360° Suture Trabeculotomy, Kahook Dual Blade, and Tanito Microhook Procedures: A Comparative Study

**DOI:** 10.3390/jcm14134610

**Published:** 2025-06-29

**Authors:** Etsuo Chihara, Tomoyuki Chihara

**Affiliations:** Sensho-kai Eye Institute, Minamiyama 50-1, Iseda, Uji, Kyoto 611-0043, Japan; mail4chiharat@gmail.com

**Keywords:** minimally invasive glaucoma surgery (MIGS), Kahook dual blade, T-hook, Tanito microhook, 360-degree suture trabeculotomy, surgical outcome

## Abstract

**Aim**: To compare the three-year surgical outcomes among the Kahook dual blade (KDB), Tanito microhook (TMH), T-hook, and 360° suture trabeculotomy (S-lot) cohorts. **Study design**: Retrospective interventional comparative study conducted at a single eye center. **Subjects and Methods**: A total of 224 eyes that underwent combined cataract surgery with either KDB, TMH, T-hook, or S-lot procedures were retrospectively analyzed over the three-year period. **Results**: According to Tukey’s multiple comparison test, postoperative intraocular pressure (IOP) in the S-lot cohort was significantly lower than in the TMH cohort from 1 month to 3 years (*p* = 0.01 to <0.001), lower than in the KDB cohort between 6 months and 1 year (*p* = 0.026 to <0.001), and lower than in the T-hook cohort at 1 month (*p* = 0.012) and from 6 to 12 months (*p* < 0.001). The survival probability of achieving ≤15 mmHg and ≤18 mmHg in the S-lot cohort was significantly better than in others by *p* < 0.001 and 0.005, respectively. At 3 months, the T-hook cohort showed significantly lower IOP than the TMH cohort (*p* = 0.029), and at 1 week, IOP was marginally lower than in the KDB (*p* = 0.063) and TMH (*p* = 0.052) cohorts, based on Dunnett’s test. However, no significant differences in postoperative IOP were observed among the three sectorial canal-opening surgery (COS) groups beyond 6 months. **Conclusions**: Among the four MIGS cohorts, S-lot provided the most substantial mid-term postoperative IOP reduction. The T-hook cohort showed marginally superior IOP reduction at 1 week compared to the KDB and TMH groups.

## 1. Introduction

Canal-opening surgeries (COSs), performed from inside the eye, are increasingly used to treat mild to moderate glaucoma.

A variety of devices, such as Trabectome, BANG (bent ab interno needle goniotomy), and others, are employed to open the Schlemm’s canal. These procedures preserve the conjunctiva, are associated with fewer severe postoperative complications, and maintain good postoperative visual acuity. Previous reports suggest that both the technique and the extent of trabecular meshwork incision may influence surgical outcomes; however, expert opinions remain divided. Some reports indicate that a wider incision results in better intraocular pressure (IOP) reduction [1,2,3], whereas others report no significant impact [4,5,6]. Wider incisions may increase the risk of hyphema [7], which may be associated with postoperative IOP spikes and potentially affect final surgical outcomes [8].

Although most cases of postoperative hyphema are resolved shortly after surgery, the design of the surgical instrument—whether hook, blade, or suture—may influence the incidence and severity of bleeding. Devices with a curved distal tip [9] or those using sutures (as in 360° trabeculotomy) may reduce the risk of damaging the posterior wall of the Schlemm’s canal and thereby minimize bleeding. However, evidence remains limited regarding how different surgical techniques affect postoperative bleeding and long-term IOP control.

In this study, we aimed to compare the mid-term surgical outcomes and complication profiles of four canal-opening procedures.

## 2. Materials and Methods

This retrospective study included patients with mild to moderate primary open-angle glaucoma (POAG), exfoliation glaucoma (XFG), or ocular hypertension (OH), all of whom were indicated for concomitant internal COS and small-incision cataract surgery. Patients who underwent stent-based procedures or mini-tube insertions were excluded.

A total of 389 eyes from 279 patients who underwent concomitant phacoemulsification, implantation of intraocular lens, and COSs between May 2018 and July 2024 at Sensho-kai Eye Institute were included. The three-year outcomes of four different COS procedures, namely, Kahook dual blade (KDB: New World Medical, Rancho Cucamonga, CA, USA, 22BAIBZX00022000 JFC), Tanito microhook (TMH: Inami, Tokyo, Japan, M2215S), T-hook (Inami, Tokyo, Japan, M-2225 and Handaya, Tokyo, Japan, HS-9939), and 360° suture trabeculotomy (S-lot: Handaya, Tokyo, Japan, HS 2756), were analyzed. The choice of device primarily depended on the timing of the introduction of these devices at Sensho-kai; KDB was selected between 2016 and 2019, TMH between 2018 and 2021, and T-hook between 2021 and 2024. The criteria for selecting sectorial COS procedures (KDB, TMH, T-hook, and S-lot) were the same. The selection of S-lot was at the discretion of one of the authors (TC), who preferred this procedure.

Preoperative intraocular pressure (IOP) was defined as the highest IOP recorded within the three months prior to surgery. Additionally, the “preoperative 3-mean IOP” was calculated as the average of three consecutive IOP measurements taken under medication before surgery.

**Inclusion criteria:** Patients were aged 40 years or older (range: 44–90 years) and had a documented preoperative IOP ≥ 18 mmHg within three months prior to surgery. If both eyes were eligible, only the right eye was included in the analysis.

**Exclusion criteria:** Patients were excluded if they met any of the following conditions:

A mean of three consecutive preoperative IOP measurements under topical medications exceeded 35 mmHg. History of prior glaucoma surgery or selective laser trabeculoplasty. Intraoperative rupture of the posterior capsule or lens luxation. Diagnosis of primary angle-closure glaucoma, secondary glaucoma, congenital glaucoma, or normal tension glaucoma. Underwent standalone COS without combined cataract surgery. Postoperative follow-up period less than 6 months.

After applying the exclusion criteria, one eye from 224 patients (43 KDB, 57 TMH, 86 T-hook, and 38 S-lot eyes) was enrolled and included in the final analysis.

Low visual acuity was converted to logMAR values according to the British conversion method [10]: counting fingers, hand motion, positive light sense, and no light sense were converted to logMAR values of 2.1, 2.4, 2.7, and 3.0, respectively.

### 2.1. Surgical Procedures

The surgical procedures have been previously reported [9]. In brief, all surgeries were performed in conjunction with cataract surgery. Following the injection of viscoelastic material and completion of anterior capsulorhexis, the devices (KDB, TMH, and T-hook) were inserted through a clear corneal opening at the 10 o’clock position, and the trabecular meshwork was incised over 120 to 150 degrees using a double-mirror Ahmed surgical goniolens (UADVX-H, Ocular, WA, USA). After completing the internal trabeculotomy, phacoemulsification, aspiration, and intraocular lens implantation were performed. After completion of cataract surgery, a 0.25% acetylcholine solution was injected into the anterior chamber, and the corneal wound was closed with a single 10-0 nylon suture. Anti-glaucoma medications were administered if postoperative IOP was elevated. Following the surgery, Gatifloxacin, 0.1% Betamethasone and 2% pilocarpine eye drops were applied four times per day for 2 to 4 weeks. The extent of canal opening was obtained from operative notes.

In the case of S-lot, a small incision of the trabecular meshwork was made using a fine slit knife after completion of capsulorhexis. A small amount of viscoelastic material was then injected into the Schlemm’s canal to expand it, and a specially designed 5-0 nylon suture adapted for suture trabeculotomy (Handaya Tokyo HS 2756) was inserted into the Schlemm’s canal. The 5-0 nylon was grasped with forceps and gently advanced to achieve 360-degree insertion into the Schlemm’s canal. If strong resistance was encountered and the nylon suture did not advance, an additional incision of the trabecular meshwork was created at a different meridian, the nylon suture was grasped at this second point, and insertion was attempted again. When the end of the suture appeared at the initial insertion site, both ends of the nylon suture were grasped and pulled to open the Schlemm’s canal.

### 2.2. Classification of Intracameral Bleedings

Post-surgical bleeding into the anterior chamber in these patients was classified using the Shimane University grading system [11], which categorizes hyphema based on severity and density, as well as the presence of clot formation. Severity of hyphema (layering) was classified into 4 categories: L0: no hyphema; L1: layered blood less than 1 mm; L2: layered blood ≥ 1 mm but not exceeding the inferior pupillary margin; L3: layered blood exceeding the inferior pupillary margin. Density of intracameral bleeding was classified into 4 categories: R0: no floating red blood cell; R1: iris patterns clearly visible despite the presence of floating red blood cells; R2: Iris patterns are not clearly visible due to floating red blood cells; R3: iris pattern completely obscured. Intracameral clot formation was classified into 2 categories: C0: no blood clot formation; C1: presence of intracameral blood clot formation.

**Statistical analysis**: Statistical analyses were performed using Bell Curve for Excel (Social Survey Research Information Co., Tokyo, Japan). Multiple comparisons were evaluated using Tukey’s and Dunnett’s tests. Kaplan–Meier survival analysis was used to assess surgical success over time. The Wilcoxon signed-rank test was employed for paired comparison; Haberman residual analysis was used for categorical data.

## 3. Results

Demographic baseline data are presented in Table 1. There was no significant difference among the cohorts in baseline age, logMAR best-corrected visual acuity, preoperative IOP, the mean of three consecutive preoperative IOP measurements under topical medications, or the number of preoperative medications. However, the refractive error in the T-hook cohort was significantly less than that in the S-lot cohort (*p* = 0.048). The mean deviation (MD) of the Humphrey Visual Field Analyzer was significantly worse in the S-lot cohort compared to the other three COS cohorts (*p* < 0.005).

The extent of canal opening in the S-lot cohort was 325 ± 87°, which was significantly greater than that in the T-hook (153 ± 52), KDB (137 ± 28), and TMH (129 ± 23°) cohorts (*p* < 0.001). The difference between T-hook and TMH (*p* = 0.067) and between T-hook and KDB (*p* = 0.43) were not statistically significant according to Tukey’s multiple comparison test.

The mean follow-up periods were as follows: KDB, 47.9 ± 16.8 months; TMH, 30.3 ± 18.9 months; T-hook, 16.0 ± 9.7 months; and S-lot, 35.1 ± 21.1 months. The follow-up period for the T-hook cohort was significantly shorter (*p* < 0.001), while that for the KDB cohort was significantly longer than the other three COS cohorts (*p* < 0.005), as determined by Tukey’s multiple comparison test.

**Figure 1:**
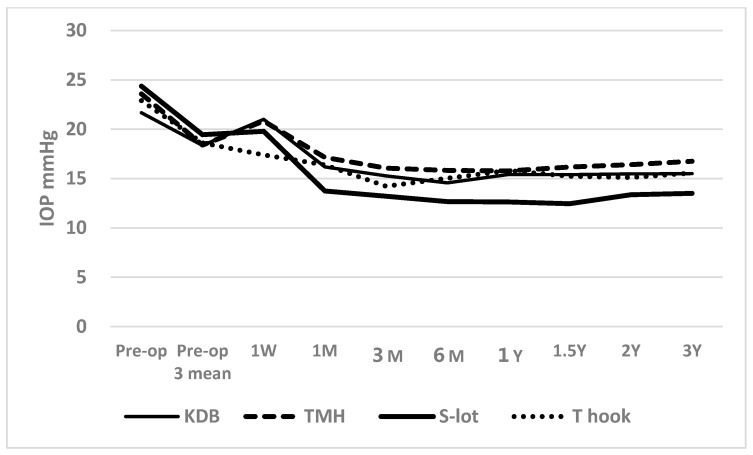
Time course of postoperative IOP in the four canal-opening surgery cohorts. A transient elevation (“hump”) in IOP was observed at 1 week postoperatively in the KDB, TMH, and S-lot cohorts, likely reflecting a response to postoperative intracameral bleeding. Following this initial rise, IOP significantly decreased in all cohorts and remained reduced through 3 years of follow-up. Postoperative IOP in the S-lot cohort was significantly lower than in the other three sectorial COS cohorts, as determined by Tukey’s multiple comparison test (Table 2).

Figure 1 shows the time course of postoperative IOP across the four COS cohorts (Figure 1).

Postoperatively, the IOP between 1 month and 3 years was significantly lower than preoperative IOP in all four cohorts, with a *p* < 0.001 using the Wilcoxon signed-rank test. A short-term elevation in IOP was observed at 1 week in the KDB, TMH, and S-lot cohorts, likely reflecting the effects of postoperative intracameral bleeding. Despite this early increase, the IOP at 1 week remained significantly lower than preoperative levels in the TMH (*p* = 0.005), S-lot (*p* = 0.011), and T-hook (*p* < 0.001) cohorts; however, the reduction in the KDB cohort was not significant (*p* = 0.198).

The number of anti-glaucoma medications significantly decreased from baseline in all cohorts. Preoperative medications used in the KDB, TMH, S-lot, and T-hook cohorts were 2.6 ± 1.3, 2.7 ± 1.5, 3.2 ± 1.1, and 2.8 ± 1.3, respectively. At 3 months postoperatively, the number of medications decreased to 1.2 ± 1.0, 1.7 ± 1.4, 2.1 ± 1.3, and 1.8 ± 1.3, respectively (all *p* < 0.001, Wilcoxon signed-rank test). A gradual increase in medication use was observed at 2 years, reaching 1.6 ± 1.2, 1.8 ± 1.3, 2.4 ± 1.4, and 2.4 ± 0.9, respectively. Despite this increase, the number of medications at two years remained significantly lower than the preoperative baseline in all cohorts (*p* < 0.05, Wilcoxon signed-rank test).

Although there was no difference in baseline preoperative IOP or the mean of three preoperative IOP measurements under medication among the four COS cohorts, one-way ANOVA revealed a significant difference in postoperative IOP among the cohorts over the follow-up period from 1 month to 3 years (*p* < 0.05, Table 2).

According to Tukey’s multiple comparison test, the postoperative IOP in the S-lot cohort was significantly lower than that in the TMH cohort at 1 month through 3 years, the KDB cohort at 6 months and 12 months, and the T-hook cohort at 1 month, 6 months, and 1 year, respectively (Table 2).

A significant difference in the percentage reduction in IOP was observed among the four COS cohorts between 3 months and 2 years, as determined by one-way ANOVA (Table 3). Using Tukey’s multiple comparison test, the percentage IOP reduction in the S-lot cohort was significantly greater than that in the KDB cohort between 3 months and 1 year, the TMH cohort between 6 months and 2 years, and the T-hook cohort between 6 months and 1 year. In contrast, no significant differences were observed in the percentage IOP reduction among the three sectorial COS procedures (KDB, TMH, and T-hook).

According to Dunnett’s multiple comparison test, the comparison between T-hook and KDB cohorts yielded a *p*-value of 0.089 at 3 months. Although the T-hook cohort showed greater IOP reduction, this difference was not statistically significant.

**Figure 2:**
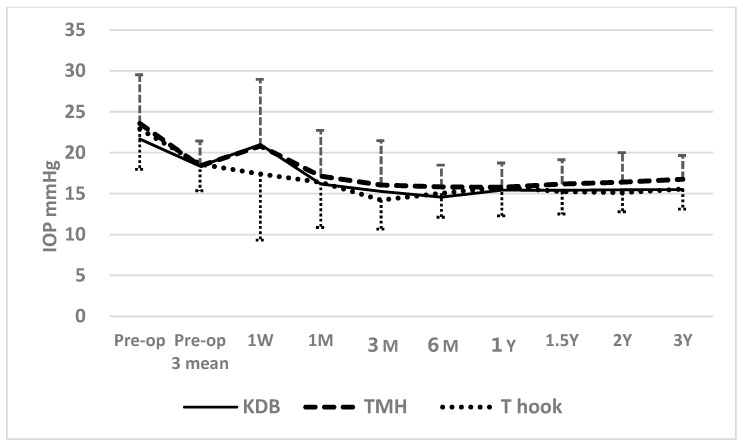
Time course of postoperative IOP in the three sectorial COS cohorts. At 1 week postoperatively, IOP in the KDB (*p* = 0.063) and TMH (*p* = 0.052) cohorts was marginally higher than in the T-hook cohort. At 3 months, IOP in the TMH cohort was significantly higher than in the T-hook cohort (*p* = 0.029; Table 2), potentially reflecting a higher prevalence of postoperative clot formation and a greater density of red blood cells in the anterior chamber (Table 4 and Table 5). However, from 6 months onward, no differences in IOP were observed among the three sectorial COS cohorts.

When comparing postoperative IOP among three sectorial COS cohorts (KDB, TMH, and T-hook), a transient elevation above the mean of three preoperative IOP measurements at 1 week was observed in KDB and TMH cohorts (Figure 2). According to Dunnett’s multiple comparison test, IOP measurements in the KDB (*p* = 0.063) and TMK (*p* = 0.052) cohorts at 1 week were marginally higher than in the T-hook cohort, possibly due to greater intracameral bleeding and clot formation in the KDB and TMH cohorts (Table 4 and Table 5). The significant difference in IOP between the T-hook cohort and TMH cohort at 3 months (*p* = 0.029), according to Dunnett’s multiple comparison test, may also reflect effects of intracameral bleedings (Table 2). After 3 months, no significant differences in IOP were detected among the three sectorial COS groups through 3 years of follow-up (Table 2).

**Figure 3:**
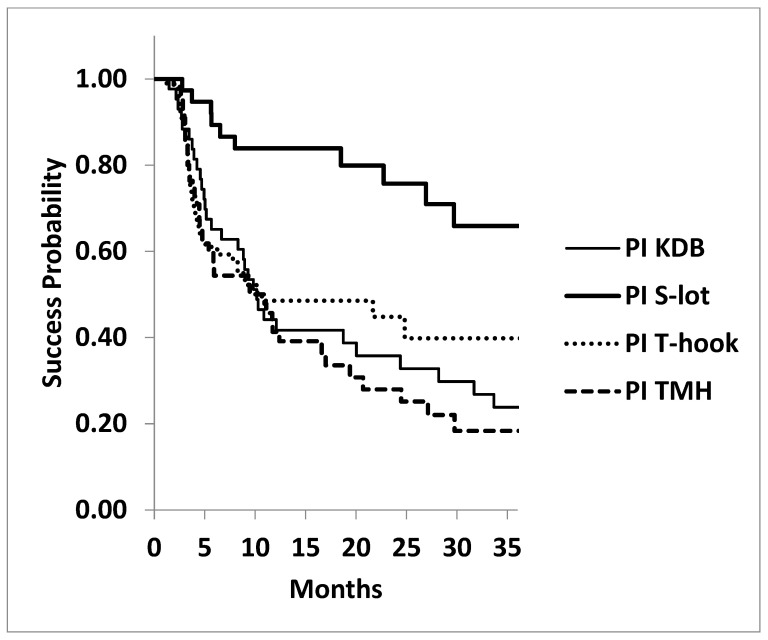
Kaplan–Meier survival curves: probability of achieving postoperative IOP ≤ 15 mmHg following four types of canal-opening, minimally invasive glaucoma surgeries (MIGSs). The cumulative success probability of achieving postoperative IOP ≤ 15 mmHg at three years was significantly higher in the 360° canal-opening procedure (S-lot cohort) compared to the three sectorial canal-opening procedures (Kahook dual blade, Tanito microhook, and T-hook) (*p* < 0.001, log-rank test). No statistically significant differences were found among the three sectorial canal-opening procedures (*p* = 0.478, log-rank test).

The Kaplan–Meier life table analysis supported these findings. A significant difference was observed among the four COS cohorts in achieving postoperative IOP targets of ≤15 mmHg and ≤18 mmHg under medication. At 3 years, cumulative success probability for achieving IOP ≤ 15 mmHg was highest in the S-lot cohort (65.9 ± 9.4%), compared to KDB (23.9 ± 7.0%), T-hook (39.9 ± 7.4%), and TMH (18.4 ± 6.4%), with a significant difference by log-rank test (*p* < 0.001; Figure 3). In contrast, no significant difference was found among the three sectorial COS procedures for this outcome (*p* = 0.478, log-rank test).

**Figure 4:**
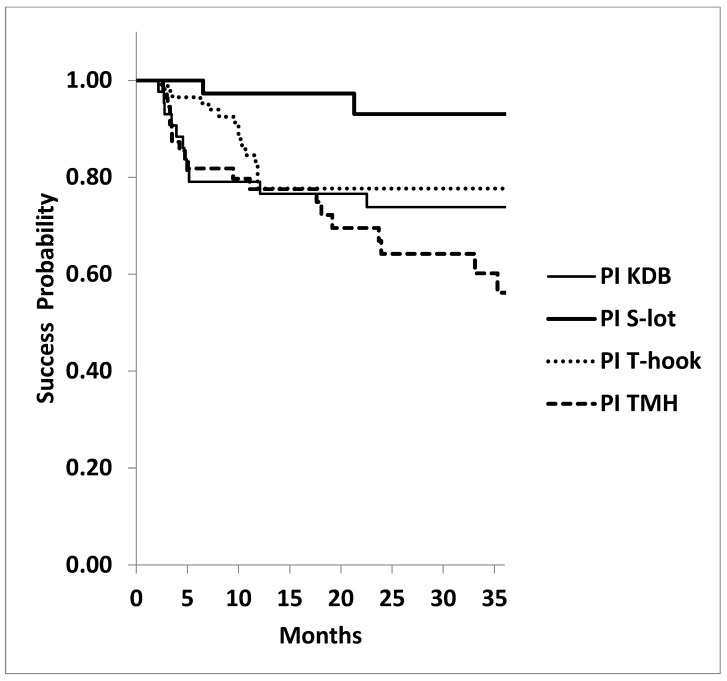
Kaplan–Meier analysis: probability of achieving postoperative IOP ≤ 18 mmHg at 3 years under medications following four types of canal-opening, minimally invasive glaucoma surgeries (MIGSs). The cumulative success probability of achieving postoperative IOP ≤ 18 mmHg at three years was again significantly higher in the 360º canal-opening procedure (S-lot cohort) compared to the other three sectorial canal-opening procedures (Kahook dual blade, Tanito microhook, and T-hook) (*p* = 0.0052, log-rank test). No significant differences in success probability were observed among the three sectorial canal-opening procedures (*p* = 0.121, log-rank test).

The success probability at three years for achieving IOP ≤ 18 mmHg under medications was also significantly higher in the S-lot cohort (93.1 ± 4.9%) compared to the KDB (73.9 ± 7.8%), T-hook (77.7 ± 5.7%), and TMH (56.2 ± 8.3%) cohorts (*p* = 0.0052, log-rank test; Figure 4). However, no significant differences were observed among the three sectorial COS procedures (*p* = 0.121, log-rank test).

The success probability for achieving ≥20% IOP reduction at three years in KDB, S-lot, T-hook, and TMH was 59.2 ± 7.7%, 78.4 ± 7.7%, 56.3 ± 10.0%, and 45.1 ± 8.1%, respectively. And the difference was marginally significant (*p* = 0.0518, log-rank test).

There was no significant difference among the four COS procedures at 3 years in achieving an IOP ≤ 21 mmHg at three years. The success probability in KDB, S-lot, T-hook, and TMH cohorts was 95.0 ± 3.4%, 93.1 ± 4.8%, 92.0 ± 3.9%, and 88.6 ± 4.4%, respectively (*p* = 0.511, log-rank test).

### 3.1. Postoperative Intracameral Bleeding

When post-surgical intracameral bleeding was compared among the four cohorts, L1 layer bleeding was observed in 36.8% of patients who underwent S-lot, which was the highest incidence among the four COS cohorts (*p* = 0.001, Haberman residual analysis; Table 6).

Blood coagula formation in the anterior chamber was observed in 19.8% of cases in the T-hook cohort, which was significantly lower than in the other three COS cohorts (*p* < 0.001). In contrast, coagula were observed in 49.1% of cases in the TMH cohort, representing the highest prevalence among the four groups (*p* = 0.004).

R0 (no floating red blood cell on the first postoperative day) was observed in 27.9% of patients who underwent the T-hook procedure, significantly more frequent than in the other cohorts (*p* < 0.001). In contrast, the R0 was rare in the TMH (*p* = 0.004) and S-lot (*p* = 0.009) cohorts. R2 (iris pattern not clearly visible due to floating red blood cells) was noted in 39.5% of S-lot cases, which was significantly higher than in other cohorts. While it was observed in only 15.1% of T-hook cases, which was significantly lower than others (*p* = 0.013).

The average time required for resolution of intracameral bleeding was shorter in the S-lot cohort at 3.1 ± 2.4 days, significantly faster than that in the KDB cohort, which required 9.1 ± 6.0 days (*p* = 0.006, Tukey’s multiple comparison test).

The resolution times for the T-hook and TMH cohorts were 6.4 ± 8.1 days and 6.3 ± 6.8 days, respectively. Differences among the KDB, T-hook, and TMH groups were not statistically significant (*p* = 0.43–1.00, multiple comparison test).

### 3.2. Postoperative IOP Spike

A postoperative spike was defined as an elevation of ≥5mmHg above the mean of three consecutive preoperative IOP measurements occurring within two weeks after surgery. The T-hook cohort had the lowest spike rate at 36.0%, which was significantly less than in the other three COS groups (*p* < 0.001; Haberman residual analysis, Table 7). In contrast, the TMH cohort had the highest spike rate at 66.7%, significantly more frequent than in other cohorts (*p* = 0.004, Table 7).

## 4. Discussion

There are several factors that may influence postoperative IOP following canal-opening surgery. One such factor is the extent of the Schlemm’s canal opening. Theoretically, a wider opening of the canal should result in greater IOP reduction [12]. However, if one or two intact aqueous veins are sufficient to drain enough aqueous humor [13], a broad canal opening may not be necessary. It is reported that active aqueous veins are located in the inferonasal quadrant of the angle [14,15], suggesting that a sectorial opening in this region alone may be sufficient to reduce IOP. Several reports suggest that an opening of 90 to 120 degrees can be sufficient for significant IOP reduction [7,16,17,18,19]. Some reports even suggest that implantation of a single iStent may achieve an IOP reduction comparable to that seen with KDB procedures [20,21]. Conversely, other studies have shown that 360° goniotomy results in greater IOP reduction than sectorial goniotomy [1,2,22,23].

Since blood coagula tend to settle in the inferior half of the eye, peripheral anterior synechia (PAS), which also commonly develops in this region, may obstruct the inferior canal opening. In this study, the mid-term IOP reduction in eyes that underwent sectorial opening of the Schlemm’s canal in the inferior quadrant was less pronounced compared to that in the S-lot cohort, where the trabecular meshwork was circumferentially opened. This finding suggests that enhanced aqueous outflow through the superior quadrant in eyes with 360° trabecular meshwork incision may have contributed to greater IOP reduction than in eyes treated with inferior sectorial incisions (KDB, TMH, and T-hook cohorts).

Postoperative intracameral bleeding may contribute to PAS formation, transient IOP spikes, and poor IOP control [8,24]. The trabecular meshwork is avascular, and injury to the trabecular meshwork is not responsible for postoperative bleeding. The main cause of postoperative bleeding is attributed to backflow from the collector channel and impairment of backyard tissue named Bell [25]. Here, the sharp tip of devices may cause penetration of the Bell and bleeding. In this study, postoperatively, the IOP at 1 week was marginally higher in the TMH and KDB cohorts compared to the T-hook cohort, in which the incidence of clot formation and the density of intracameral bleeding were significantly lower. As shown in Figure 2, the IOP at 1 week in the KDB and TMH cohorts exceeded the mean of three consecutive preoperative IOP measurements, whereas the T-hook cohort showed a lower IOP. These findings suggest that the higher incidence of clot formation and intracameral bleeding may have contributed to the transient IOP elevation observed in KDB and TMH cohorts at 1 week. Despite the short-term IOP rise in KDB and TMH cohorts at 1 week and the higher IOP in the TMH cohort at 3 months, no significant differences in postoperative IOP were observed among the three sectorial COS groups after 6 months. This finding suggests that the impact of postoperative bleeding on IOP is transient and does not persist long-term.

In the S-lot cohort, postoperative IOP elevation at 1 week was 19.9 ± 10.0, which was milder than that observed in the KDB and TMH cohorts (Table 2). The intracameral bleeding resolved in an average of only 3.1 days, which was shorter than in the other cohorts. This suggests that the wide circumferential opening of the trabecular meshwork may have facilitated efficient clearance of red blood cells from the anterior chamber.

Another potential confounding factor affecting surgical outcome is the wound-healing response at the trabecular meshwork. Suture trabeculotomy does not involve excision of the trabecular meshwork, whereas KDB excises the meshwork tissue. In contrast, TMH and T-hook do not remove the trabecular meshwork but instead displace it to create a “double door” opening. Despite these differences in the mechanism of canal opening, previous studies comparing KDB, TMH, and T-hook have not demonstrated significant differences in surgical outcomes [26,27]. The findings of the current study similarly suggest that, in terms of mid-term outcomes, there is no significant difference in IOP reduction among the three sectorial COS procedures.

Therefore, excision of the trabecular meshwork tissue may not be essential in achieving mid-term IOP reduction.

Although postoperative bleeding typically resolves shortly after surgery in most cases, severe complications such as corneal blood staining can occasionally occur. In case of massive hyphema accompanied by intense pain, surgical interventions such as paracentesis and anterior chamber washout may be required. The lower incidence of postoperative bleeding and IOP spikes in the T-hook cohort suggests that the use of the T-hook may offer clinical benefits for patients.

The T-hook features blades on both sides of the shaft, allowing it to bilaterally incise the trabecular meshwork. Compared to other canal-opening surgery (COS) devices, it has the advantage of enabling a broader incision with a single insertion into the anterior chamber. Furthermore, the rounded distal tip minimizes the risk of damaging the outer wall of the Schlemm’s canal, thereby reducing the likelihood of traumatic bleeding.

**Figure 5:**
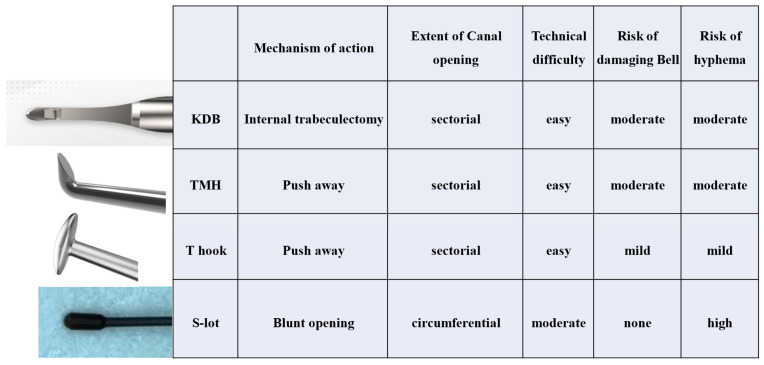
Designs of four devices and brief summary of relevant complications.

In Figure 5, we summarized features of four devices and a brief summary of relevant complications.

In this study, we included POAG, XFG, and OH for analysis. XFG is characterized by higher IOP, faster visual field deterioration, and a poor response to medical therapy. Several authors have reported that IOP reduction in XFG achieved through canal-opening surgery surpasses that observed in POAG [28,29], whereas others have reported equivalent IOP reduction between XFG and POAG [30]. Therefore, the potential difference in surgical response between XFG and POAG may warrant further investigation in future studies.

Another confounding factor is the effects of combined cataract surgery. Many reports have shown that the combination of cataract and canal-opening surgery (COS) provides additional benefit in IOP reduction [1,31]. However, several authors have reported no enhanced IOP reduction with combined cataract and COS compared to COS alone [17]. In this study, we studied outcomes exclusively in cases of combined surgery, so the effects of cataract surgery will not influence the results.

## 5. Conclusions

Postoperative IOP reduction was compared among four canal-opening MIGS procedures. The most significant IOP reduction was observed in the S-lot cohort.

The T-hook cohort showed the least postoperative intracameral bleeding. While intracameral bleeding in the KDB and TMH cohorts was associated with short-term IOP elevation, it did not impact long-term IOP outcomes between 6 months and 3 years.

## 6. Limitation of This Study

This study is retrospective and non-randomized. Although preoperative IOP was comparable among cohorts, the S-lot cohort exhibited significantly more advanced visual field defects, suggesting that surgeons may have preferentially selected S-lot for more severe glaucoma cases. This could represent a potential bias that may have affected the outcomes. Randomized selection of surgical procedure is desirable for future studies. Additionally, the follow-up period for the T-hook cohort was significantly shorter, likely due to the recent introduction of the device.

To allow for a more accurate and unbiased comparison of surgical procedures, future studies should adopt a prospective, randomized design.

## Figures and Tables

**Table 1:** Baseline characteristics of the four canal-opening surgery cohorts.

	Age	logMAR	Preop IOP	Preop 3 Mean IOP	# Pre-meds	SQ Ref	MD	Extent of Canal Opening
KDB	72.9 ± 8.6	0.186 ± 0.369	21.7 ± 3.6	18.4 ± 2.9	2.6 ± 1.3	−3.5 ± 4.7	−9.45 ± 8.12	137 ± 28
TMH	68.9 ± 10.9	0.115 ± 0.244	23.6 ± 6.0	18.4 ± 3.1	2.7 ± 1.5	−4.1 ± 4.8	−10.38 ± 8.59	129 ± 23
S-lot	73.0 ± 8.5	0.154 ± 0.477	24.4 ± 9.2	19.4 ± 5.8	3.2 ± 1.1	−4.5 ± 4.5	−16.92 ± 8.48	325 ± 87
T-hook	70.9 ± 8.0	0.110 ± 0.288	22.9 ± 5.0	18.6 ± 3.3	2.8 ± 1.3	−2.1 ± 4.2	−9.96 ± 7.35	153 ± 53
*p* by one-way ANOVA	0.076	0.618	0.201	0.519	0.143	0.069	<0.001	<0.001
Significant difference by Tukey’s test	NS	NS	NS	NS	NS	T-hook vs. S-lot *p* = 0.048	S-lot vs. other 3 COS *p* < 0.005	S-lot vs. 3 other COS *p* < 0.001

IOP: intraocular pressure, # pre-med: number of preoperative medications, SQ ref: spherical equivalent refractive error, MD: mean deviation using Humphrey Visual Field Analyzer, KDB: concomitant cataract surgery with Kahook dual blade, TMH: concomitant cataract surgery with Tanito microhook, S-lot: concomitant cataract surgery with 360° suture trabeculotomy, T-hook: concomitant cataract surgery with T-hook, ANOVA: analysis of variance, NS: not significant, COS: canal-opening surgery.

**Table 2:** Time course of IOP after four different COS procedures, and differences among them assessed by multiple comparison test.

		Pre-IOP	Mean of 3 Pre-op IOPs	1 W	1 M	3 M	6 M	1 Y	2 Y	3 Y
	KDB	21.8 ± 3.6	18.4 ± 3.0	20.5 ± 8.8	16.3 ± 4.1	15.3 ± 4.2	14.6 ± 3.5	15.4 ± 3.1	15.5 ± 3.4	15.5 ± 3.0
	TMH	23.5 ± 6.0	18.3 ± 3.0	20.5 ± 7.8	16.6 ± 3.1	15.7 ± 3.8	15.8 ± 2.7	15.8 ± 3.0	16.4 ± 3.7	16.8 ± 3.0
	T-hook	22.9 ± 5.0	18.6 ± 3.3	17.4 ± 8.1	16.4 ± 5.6	14.2 ± 3.6	15.0 ± 2.9	15.8 ± 3.6	15.1 ± 2.4	15.6 ± 2.6
	S-lot	24.5 ± 9.3	19.6 ± 5.8	19.9 ± 10.0	13.6 ± 4.3	13.2 ± 2.6	12.7 ± 2.7	12.6 ± 2.7	13.4 ± 2.9	13.5 ± 3.4
	*p* by one-way ANOVA	0.238	0.413	0.101	0.01	0.006	<0.001	<0.001	0.006	0.021
*p*-value by Tukey’s test	Significant difference noted between specific cohorts by Tukey’s test	NS	NS	NS	S-lot vs. T-hook *p* = 0.012; S-lot vs. TMH *p* = 0.012	S-lot vs. TMH *p* = 0.008	S-lot vs. TMH and T-hook *p* < 0.001; S-lot vs. KDB *p* = 0.026	S-lot vs. KDB, TMH, T-hook all *p* < 0.001	S-lot vs. TMH *p* = 0.003	S-lot vs. TMH *p* = 0.01
	Marginal difference noted by Tukey’s test	NS	NS	NS	S-lot vs. KDB *p* = 0.055	S-lot vs. KDB *p* = 0.057			S-lot vs. KDB *p* = 0.074	
*p*-value by Dunnet’s test	Between S-lot and KDB cohort	0.057	0.183	0.818	0.015 *	0.016 *	0.007 **	<0.001	0.020 *	0.04 *
	Between S-lot and TMH cohort	0.381	0.125	0.814	0.003 **	0.002 **	<0.001	<0.001	<0.001	0.003 **
	Between S-lot and T-hook cohort	0.179	0.185	0.139	0.003 **	0.165	<0.001	<0.001	0.07	0.124
	Between T-hook and KDB cohort	0.333	0.68	0.063	0.711	0.196	0.986	0.509	0.872	0.668
	Between T-hook and TMH cohort	0.934	0.623	0.052	0.874	0.029 *	0.996	0.745	0.993	0.938

*: *p* < 0.05, ** *p* < 0.01.

**Table 3:** Comparison of percentage IOP reduction among four COS procedures and statistical differences assessed by multiple comparison tests.

		% IOP Reduction at 3 M		% IOP Reduction at 6 M		% IOP Reduction at 1 Y		% IOP Reduction at 2 Y		% IOP Reduction at 3 Y	
type		n	mean ± SD	n	mean ± SD	n	mean ± SD	n	mean ± SD		mean ± SD
KDB		41	29.1 ± 18.0	41	31.9 ± 15.7	41	27.7 ± 14.9	37	27.9 ± 15.3	36	27.0 ± 18.2
TMH		55	31.3 ± 16.2	55	30.3 ± 14.2	50	31.6 ± 15.7	42	27.8 ± 15.7	20	23.7 ± 16.6
T-hook		86	35.7 ± 18.8	86	30.9 ± 18.6	50	29.5 ± 17.0	27	33.6 ± 15.4	9	30.6 ± 22.6
S-lot		37	39.9 ± 20.3	37	42.9 ± 17.9	35	41.9 ± 20.1	22	39.3 ± 19.7	16	36.7±22.5
*p* by one-way ANOVA			0.038 *		0.002 *		0.002 *		0.029 *		0.230
Level 1	Level 2	Difference	*p* by Tukey	Difference	*p* by Tukey	Difference	*p* by Tukey	Difference	*p* by Tukey	Difference	*p* by Tukey
KDB	TMH	2.2	0.934	1.6	0.968	3.9	0.692	0.2	1.000	3.3	0.922
KDB	T-hook	6.5	0.231	1.0	0.988	1.8	0.959	5.7	0.511	3.6	0.955
KDB	S-lot	10.7	0.048 *	11.0	0.022 *	14.1	0.002 *	11.3	0.051	9.6	0.333
TMH	T-hook	4.3	0.511	0.6	0.997	2.1	0.922	5.8	0.465	6.9	0.796
TMH	S-lot	8.5	0.124	12.6	0.003 *	10.3	0.031 *	11.5	0.040 *	12.9	0.185
T-hook	S-lot	4.2	0.639	12.1	0.002 *	12.4	0.006 *	5.7	0.614	6.0	0.868

M: months, Y: years, n: sample size, SD: standard deviation, Tukey: Tukey’s multiple comparison test. *: *p* < 0.05.

**Table 4:** Prevalence of intracameral blood coagula formation in each COS cohort.

	C0	C1	*p* by Adjusted Standardized Residual
KDB N = 43	67.4%	32.6%	0.886
TMH N = 57	50.9%	49.1%	0.004 *
S-lot N = 38	57.9%	42.1%	0.216
T-hook N = 86	80.2%	19.8%	*p* < 0.001 *

*: *p* < 0.05.

**Table 5:** Density of intracameral bleeding the next day after surgery.

	Density of Intracameral Bleeding				*p* by Adjusted Standardized Residual			
	R0	R1	R2	R3	R0	R1	R2	R3
KDB	9.3%	53.5%	23.3%	14.0%	0.428	0.854	0.885	0.445
TMH	1.8%	56.1%	28.1%	14.0%	0.004 *	0.494	0.418	0.348
S-lot	0%	50.0%	39.5%	10.5%	0.009 *	0.762	0.015 *	0.967
T-hook	27.9%	50.0%	15.1%	7.0%	<0.001 *	0.598	0.013 *	0.153

*: *p* < 0.05.

**Table 6:** Difference in incidence of intracameral layer bleeding among four COS cohorts.

	Prevalence of Layered Bleeding (%)				*p* by Adjusted Standardized Residual			
	L0	L1	L2	L3	L0	L1	L2	L3
KDB N = 43	67.4	11.6	16.3	4.7	0.285	0.208	0.828	0.814
TMH N = 57	57.9	15.8	21.1	5.3	0.672	0.570	0.401	0.579
S-lot N = 38	42.1	36.8	15.8	5.3	0.012 *	0.001 *	0.772	0.668
T-hook N = 86	66.3	15.1	16.3	2.3	0.147	0.330	0.724	0.309

*: *p* < 0.05.

**Table 7:** Prevalence of postoperative IOP spike in each COS cohort.

	Prevalence of Spike Exceeding 5 mmHg		*p* by Adjusted Standardized Residual
	Spike+	Spike−	
KDB	62.8%	37.2%	0.062
TMH	66.7%	33.3%	0.004 *
S-lot	42.1%	57.9%	0.285
T-hook	36.0%	64.0%	<0.001 *

*: *p* < 0.05.

## Data Availability

The research data used in this research is available upon request from the corresponding author.

## Abbreviations

The following abbreviations are used in this manuscript:

KDB	Kahook dual blade
TMH	Tanito microhook
S-lot	360° suture trabeculotomy
COS	Canal-opening surgery
MIGS	Minimally invasive glaucoma surgery
PAS	Peripheral anterior synechia
